# Pre-Sleep Consumption of Casein and Whey Protein: Effects on Morning Metabolism and Resistance Exercise Performance in Active Women

**DOI:** 10.3390/nu10091273

**Published:** 2018-09-10

**Authors:** Takudzwa A. Madzima, Jared T. Melanson, Jonas R. Black, Svetlana Nepocatych

**Affiliations:** Department of Exercise Science, Energy Metabolism and Body Composition Laboratory, Elon University, 100 Campus Drive, Elon, NC 27244, USA; jmelanson@elon.edu (J.T.M.); jblack14@elon.edu (J.R.B.); snepocatych@elon.edu (S.N.)

**Keywords:** pre-sleep feeding, whey protein, casein protein, metabolism, resistance exercise

## Abstract

Consuming milk proteins (casein (CP) and whey (WP)) at night before sleep has been shown to positively influence next morning resting metabolic rate (RMR). No data exist regarding the effect of pre-sleep consumption of CP and WP on the ability to perform resistance exercise (RE) the following morning. The present study compared the effects of low (24 g) and high (48 g) doses of CP and WP and a non-energetic placebo (PLA) consumed 30 min before sleep on morning RMR, and RE performance. Nine active women participated in this randomized, double-blind, crossover study. Next morning RMR was measured via indirect calorimetry. RE was performed on six machines for 2 sets of 10 repetitions, and a 3rd set to failure at 60% of one-repetition maximum to calculate RE volume (weight lifted × sets × repetitions). Magnitude based inferences were used. Compared to the PLA, 48 g CP had a likely increase in RMR (4.0 ± 4.8%) and possibly trivial (1.1 ± 7.0%) effect on RE volume. There were no clear effects of 24 g CP, 24 g and 48 g of WP on RMR and RE volume. In conclusion, 48 g CP elicited favorable changes in morning RMR, with only trivial changes in RE performance.

## 1. Introduction

Pre-sleep protein feeding within 30 min of sleep has been posited as a new window of opportunity in nutrient timing research [1], conferring benefits including increased next morning resting metabolic rate (RMR) [2], and overnight muscle protein synthesis (MPS) and recovery [3,4]. Previous concerns about pre-sleep feeding have been related to the belief that eating late at night leads to weight gain [5]. While the concern of weight gain is understandable as RMR is lower overnight [6], recent pre-sleep feeding studies have shown that next morning RMR was increased [2,7] or unhindered [8,9] after consumption of low energy (~600 kJ; 150 kcals), protein dense foods prior to sleep. Improvements in morning RMR may have an impact on total daily energy expenditure, which may help individuals seeking to maintain or improve body composition.

Commonly consumed pre-sleep snacks include the milk proteins casein (CP), and whey (WP), with CP often recommended as the pre-sleep protein type for active individuals [3]. The acidic environment of the stomach causes CP to clot, thereby delaying the gastric emptying into the small intestine resulting in a moderate, sustained increase in plasma amino acid concentrations [10]. On the other hand, WP is acid-soluble and empties into the small intestine more rapidly, resulting in a more pronounced but temporal rise in plasma amino acid levels [10,11]. Thus, CP may be a more ideal protein type to consume prior to sleep, which will prolong overnight hyperaminoacidemia and provide the precursors for overnight protein metabolism, thereby increasing RMR. However, it is inconclusive whether CP is superior to WP, as daytime studies observed no differences in postprandial RMR between isoenergetic (1465 kJ; 350 kcals) mixed breakfast meals, each containing either 34 g CP or 36 g WP [12]. In contrast, a mixed meal containing WP increased RMR more than isoenergetic (1921 kJ; 459 kcals) CP in lean men and women [13]. 

When consumed in close proximity to sleep, a recent acute study by Madzima et al. [2] found similar increases in next morning RMR after consuming 30 g of both CP and WP prior to sleep in active men, when compared to a non-energetic placebo (PLA). Interestingly, next morning fat oxidation was greater after the PLA compared to WP, but not CP. In addition, Kinsey et al. [8,9] did not observe an advantage of consuming 30 g of CP over 30 g of WP in obese women, nor 30 g of CP compared to a PLA in obese men. Following the reported lack of differences between CP and WP, a more recent acute pre-sleep feeding study sought to investigate the impact of 10 g and 30 g protein in the form of milk, which constitutes 80% CP and 20% WP, on next morning RMR in moderately overweight men, and did not find any changes in RMR when compared to a non-caloric placebo [14]. Therefore, the composition and dose of an optimal pre-sleep meal to alter morning RMR is yet to be elucidated. Further, current evidence suggests that the only increases in next morning RMR after pre-sleep consumption of single macronutrients or low energy snacks (e.g., milk), have been observed in physically active men [2] and women [7]. Thus, a reasonable follow-up question is to determine whether the increases in RMR and protein type consumed prior to sleep have an impact on performance during a morning exercise bout in active individuals. 

Although CP has been the most extensively studied pre-sleep protein [4,8,15], it is yet to be determined whether CP is superior. WP not only contains a greater essential amino acid content than CP, but also has a higher proportion of leucine, the branched chain amino acid important for stimulating MPS [16,17]. In addition, over a 6 h postprandial period during daytime protein feeding studies, WP has been reported to stimulate MPS more than CP [18], but it is yet to be established whether this remains true during an overnight period, which can last approximately 8–10 h. It is plausible that the timeline for benefits conferred from protein consumption during waking hours, may be extended during the overnight postprandial period when individuals are asleep. Previously, MPS has not been augmented during the overnight period when 20–25 g CP was consumed during an evening exercise bout [19]; however, 40 g of CP administered immediately prior to sleep was properly digested and absorbed and increased overnight MPS and recovery in active individuals, compared to a placebo [4]. Further, pre-sleep protein augmented overnight MPS when a prior evening session of resistance exercise was performed [15]. Thus, pre-sleep protein may be a strategy to improve overnight recovery from an evening session of exercise and potentially improve performance if exercise was performed early the following morning. It should be noted that overnight MPS in the study by Trommelen et al. [15], was also stimulated after pre-sleep protein consumption alone. Therefore, it is possible that even in the absence of an evening exercise session, consumption of a bolus of protein prior to sleep will acutely stimulate overnight MPS, although not to the extent as if an evening exercise session had been performed prior. 

It is not uncommon for active individuals to perform morning exercise in a fasted state for several reasons, including seeking to avoid gastrointestinal distress, availability of time, or intending to increase fat oxidation [20,21,22]. The period between dinner and breakfast is the longest post-absorptive period during the day, which could result in >8 h without provision of nutrients. Although this length of time without nutrition may enhance next morning fat oxidation [20,23,24], it may ultimately hinder performance in individuals seeking to exercise in the early morning [25]. Consumption of a pre-sleep protein supplement may be a feeding opportunity to provide nutrients to improve overnight recovery from activities of daily living in active individuals, compared to not consuming any energy after their evening meal. 

To date, only one study has investigated the effect of pre-sleep feeding on next morning exercise performance in competitive female runners [7]. Although, Ormsbee et al. [7] reported that a pre-sleep, low-calorie (~753 kJ; 180 kcals), protein-rich source in the form of chocolate milk had a likely small increase in next morning RMR, there was no improvement in aerobic performance compared to a non-caloric placebo. Therefore, it is unclear whether pre-sleep feeding influences next morning exercise performance. Furthermore, determining the optimal dose of pre-sleep protein consumption that will increase next morning RMR, fat oxidation, and exercise performance may be beneficial for active individuals seeking to exercise early in the morning. Our previous pre-sleep feeding study [2] has shown increased next morning RMR, following a 30 g dose of pre-sleep protein. However, to the best of our knowledge, no acute pre-sleep feeding studies have investigated whether a single dose of CP or WP less than or more than the typical 30 g dose, elicits similar increases in RMR and possibly fat oxidation. Further, no acute studies have investigated the effect of pre-sleep protein on next morning resistance exercise (RE) performance. Therefore, the primary purpose of the present study was to investigate whether pre-sleep consumption of a low dose (24 g) and a high dose (48 g) of CP and WP increases next morning RMR, fat oxidation, and RE performance, when compared to a non-energetic placebo (PLA). We hypothesize that pre-sleep consumption of protein (CP or WP) would be superior in increasing next morning RMR, fat oxidation, and total volume performed during an RE bout, when compared to a PLA (which will simulate not eating anything before bed the night before). The rationale for our hypothesized increase in morning total RE volume performed, after pre-sleep protein feeding, is based on the improvement in overnight recovery from pre-sleep feeding reported in the aforementioned studies. A secondary aim of the present study was to determine any differences between CP and WP, and whether there is a dose response of CP and WP on the aforementioned variables. Owing to moderate, sustained rise in plasma amino acid concentrations after CP consumption [10], we hypothesize that pre-sleep CP will result in a greater increase in next morning RMR, fat oxidation, and volume of RE performed, compared to WP. We also hypothesize that pre-sleep consumption of the high dose of CP and WP, would be superior to the low doses of each respective protein. 

## 2. Materials and Methods 

### 2.1. Participants

Nine physically active young women, (age: 25 ± 6.4 years; body fat: 22.0 ± 6.2%; BMI 22.6 ± 2.4 kg/m^2^) participated. To be eligible, participants had to have regularly resistance trained ≥2 days per week for 12 months. Participants were excluded if they had uncontrolled hypertension (blood pressure (BP) > 160/100 mmHg), taking BP or cholesterol medications, had been diagnosed with cardiovascular disease, stroke, diabetes, thyroid, kidney dysfunction, or had dairy allergies. In addition, participants were excluded if they were currently a smoker. If they were consuming any nutritional supplements (except for a multivitamin), they were asked to refrain from taking the supplements two weeks before their first visit and during the entire study period. Participants were asked to maintain their normal exercise regimens for the duration of the study. The present study was conducted according to the guidelines laid down in the Declaration of Helsinki, and all procedures involving human participants were approved by the University Institutional Review Board. Written informed consent was obtained before participation in the study. 

### 2.2. Study Design

**Figure 1:**
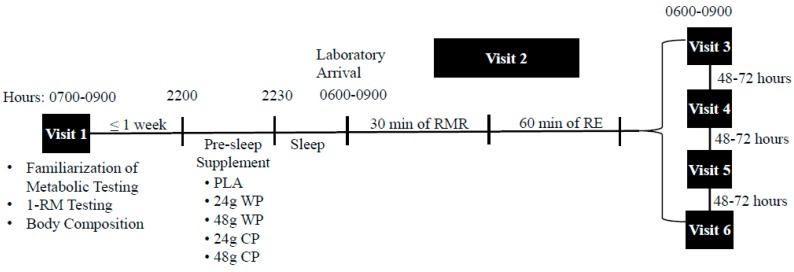
Study Timeline. 1-RM, one-repetition strength testing; RMR, resting metabolic rate measurements; the morning after pre-sleep consumption of a single serving of 24 or 48 g whey protein (WP), 24 or 28 g casein protein (CP), and a non-energetic placebo (PLA).

The present study was a randomized double-blinded crossover trial. This study included one familiarization visit and 5 experimental trials, separated by 48–72 h (Figure 1). Participants were randomly assigned using a computer generated randomization program to consume one of five supplements the night prior to each experimental trial: (1) 24 g WP (31 g, 502 kJ (120 kcals), 24 g protein, 4 g carbohydrate, 1 g fat); (2) 48 g WP (62 g, 1004 kJ (240 kcals), 48 g protein, 8 g carbohydrate, 2 g fat; (3) 24 g CP: 33 g, 502 kJ (120 kcals), 24 g protein, 4 g carbohydrate, 1 g fat; (4) 48 g CP: 66 g, 1004 kJ (240 kcals), 48 g protein, 8 g carbohydrate, 2 g fat; (5) PLA: Propel Zero™ (The Gatorade Company, Chicago, IL, USA), 2.9 g, 0 kJ (0 kcals) (placebo). The rationale for the use of the 24 g dosage for the present study, was to match typical serving sizes of most commercially available protein supplements (24–25 g per serving). Further, we sought to investigate the effect of a double serving of protein on the dependent variables. Each protein powder (Optimum Nutrition^®^, Aurora, IL, USA) was identically flavored (vanilla) and had similar texture, however the PLA that was also in powdered form had a different texture and flavor. Thus, only the PLA was single-blinded to the participants. Participants were provided each supplement in a Ziplock^®^ plastic bag (S. C. Johnson & Son, Racine, WI, USA) and instructed to consume each supplement with 12 oz of water at least two hours after dinner, and within 30 min before sleep. To confirm compliance with time of supplement consumption, participants were asked to complete a log documenting the time they completed the supplement and the time they lay down to sleep. Prior to each experimental trial, participants were asked to refrain from alcohol, caffeine, and exercise for 24 h. In addition, each participant was asked to record their dietary intake for the 24 h period prior to each experimental trial using the nutrient tracking smartphone application, MyFitnessPal^®^ (Under Armour Inc., Baltimore, MD, USA). Participants reported to the laboratory for each experimental trial the morning after consuming their assigned pre-sleep supplements, upon waking, and in a fasted state between 0600 and 0900 h. 

### 2.3. Laboratory Visit 1: Baseline Testing and Familiarization

Height and weight was assessed using a stadiometer and standardized scale (Detecto^®^, Webb City, MO, USA), respectively. Body composition was assessed via bioelectrical impedance analysis (BIA 450, Biodynamics Corp, Shoreline, WA). Upon completion of body composition assessments, one-repetition maximum (1-RM) tests were performed on the following exercise machines (Precor^®^, Woodinville, WA, USA): Chest press, leg press, lat pull-down, shoulder press, leg extension, and leg curl. After a warm-up, participants progressed towards the maximum weight that they could lift one time through a full range of motion. All measurements were recorded, with the goal of achieving a 1-RM within 3 to 5 attempts. Their achieved 1-RM was used to calculate 60% of their 1-RM, which was the load used for testing days. Participants were then familiarized with the ventilated hood (ParvoMedics, Sandy, UT, USA) to be used for metabolic testing by laying under the hood for several minutes until they felt accustomed to it. Upon completion of familiarization, participants were provided with their first supplement and instructed to take it the night before their first testing day (at least 48 h) later. 

### 2.4. Experimental Trials (Visits 2, 3, 4 and 5)

#### Metabolic Testing

Upon arrival to the laboratory, participants lay supine in a quiet, dark, and climate-controlled room (20–23 °C), whilst gas exchange was measured. Gas exchange was measured continuously for 30 min to assess resting oxygen consumption (VO_2_; mL/kg per min), RMR (kJ/day), and respiratory exchange ratio (RER) via indirect calorimetry using the ventilated hood connected to a metabolic cart (ParvoMedics, TrueOne 2400, Sandy, UT, USA). Metabolic data were averaged every 30 s, and the last 20 min were used for analysis. The RER measurements were used to determine substrate utilization of fat (RER = 0.7; 100% fat) and carbohydrate (RER = 1.0; 100% carbohydrate) as a fuel source the morning, after pre-sleep protein or PLA consumption. 

### 2.5. Resistance Exercise Performance Testing

Following metabolic testing, participants were asked to perform RE at 60% of their 1-RM, for two sets of 10 repetitions, and a third set to muscular failure on the following exercise machines: Chest press, leg press, lat pull-down, shoulder press, leg extension, and leg curl. To standardize time under tension and minimize the variation in repetition speed between experimental trials, repetitions were performed at a metronome cadence of 30 beats per minute, which was equated to a 2-s concentric and 2-s eccentric phase. Total RE volume performed was calculated by multiplying the weight lifted by 3 sets and by the number of repetitions performed. 

### 2.6. Statistical Analyses 

Probabilistic magnitude-based inferences and 90% confidence intervals (CI) were used to assess the effect of pre-sleep WP and CP on the metabolic variables and RE volume, when compared to the PLA using the methods detailed by Batterham and Hopkins [26]. Several performance and sports nutrition studies [7,27,28] have used this approach as an alternative to traditional null hypothesis testing. A published spreadsheet was used to assess the likelihood of a true treatment effect, based on the smallest meaningful threshold [29]. The smallest meaningful treatment effect thresholds were determined by multiplying 0.2 and 0.3, by the back-transformed SD as a percent of the mean of the PLA (control condition) for metabolic and performance variables, respectively [30]. Inferences were based on the spread of the 90% CI, in relation to the threshold values. An effect was classified as unclear if the 90% confidence interval overlapped both the positive and negative thresholds. The clinical version of magnitude-based inferences was used to determine the clear effects of the treatment as having a >25% chance of benefit and <0.5% chance of harm. Qualitative inferences were based on the chance of benefit (increase) and harm (decrease) of each supplement on the outcome variables, compared to the PLA as follows: <1%, almost certainly none; 1–5%, very unlikely; 5–25% unlikely; 25–75%, possible; 75–95%, likely; 95–99%, very likely; and >99%, almost certainly. Effect sizes (ES) were calculated by standardizing the differences of all treatments to the SD of the PLA; and to control for small sample bias, the SD of the PLA was divided by 1–3(4v-1), where v is equal to the degrees of freedom [30]. The ES magnitudes for metabolic variables were qualified as follows: trivial, 0.0–0.2; small, 0.2–0.6; moderate, 0.6–1.2; large, 1.2–2.0; very large, 2.0–4.0; and extremely large, >4.0. For RE performance, ES was qualified as follows: trivial, 0.0–0.3 (4%); small, 0.3–0.9 (18%); moderate, 0.9–1.6 (32%); large, 1.6–2.5 (50%); very large, 2.5–4.0 (80%); and extremely large, >4.0 [30]. Data were log-transformed to account for heteroscedastic error [31]. In addition, within-subjects repeated measures ANOVA was conducted to measure differences for metabolic variables and RE volume for each trial. When appropriate, a Tukey’s post hoc analysis was used to examine differences. Statistical analyses were performed using SPSS software (Ver. 24; IBM-SPSS Inc., Armonk, NY, USA). Significance was set at *p* < 0.05. Data presented were back-transformed means ± SD and/or mean difference (%) ± CI. Qualitative inferences were presented along associated *p*-values.

## 3. Results

### 3.1. Dietary Intake 

Analysis of pre-experimental trial 24 h dietary logs showed dietary intake was similar between trials, with a mean intake of 5933 ± 866 kJ/day (1418 ± 207 kcals/day), with 48.0 ± 3.4% energy from carbohydrates, 17.9 ± 3.1% energy from protein, and 34.1 ± 5.7% energy from fat. 

### 3.2. Metabolism

Mean VO_2_ (ml/kg per min), RMR (kJ/day), and RER, the morning after pre-sleep CP and WP, as well as the mean effect differences when compared to PLA, are presented in Table 1. With 48 g CP, there was a possibly and likely (70%, 84% likelihood, respectively) increase in VO_2_ (ES = 0.31; *p* = 0.375) and RMR; (ES = 0.43; *p* = 0.119), respectively, when compared to PLA, with no clear effect of 24 g of CP. There were no clear effects of 48 g CP on RER; however, 24 g CP elicited a possibly (70% likelihood) lower fat oxidation than PLA (ES = 0.44; *p* = 0.397). There were no clear effects of 24 g and 48 g of WP on VO_2_ and RMR. However, RER measures revealed a likely (76% likelihood) lower fat oxidation (increase in carbohydrate utilization), following 48 g WP, compared to PLA (ES = −0.53; *p* = 0.327). 

Comparison between protein type and dose are displayed in Table 2. When compared to 48 g WP, pre-sleep consumption of 48 g CP was likely beneficial (91%, 93%, 81% likelihood, respectively) to increasing VO_2_ (ES = 0.72; *p* = 0.128), RMR (ES = 0.61; *p* = 0.066) and fat oxidation (ES = 0.64; *p* = 0.241). Whereas, differences in VO_2_ and RMR were unclear between 24 g CP and 24 g WP, with 24 g CP having a likely (95% likelihood) lower fat oxidation, compared to 24 g WP (ES = −0.65; *p* = 0.040). Comparison of protein dose within protein type revealed that 48 g CP had a likely (85%, 86% likelihood, respectively) increase in VO_2_ (ES = 0.67; *p* = 0.231) and RMR (ES = 0.65; *p* = 0.163), whilst RER remained unclear. Mean differences between 48 g WP and 24 g WP were unclear for VO_2_, whilst 48 g WP possibly (56% likelihood) increased RMR (ES = 0.25; *p* = 0.651) and very likely (96% likelihood) decreased fat oxidation (ES = −0.74; *p* = 0.033). 

### 3.3. Resistance Exercise Performance 

**Figure 2:**
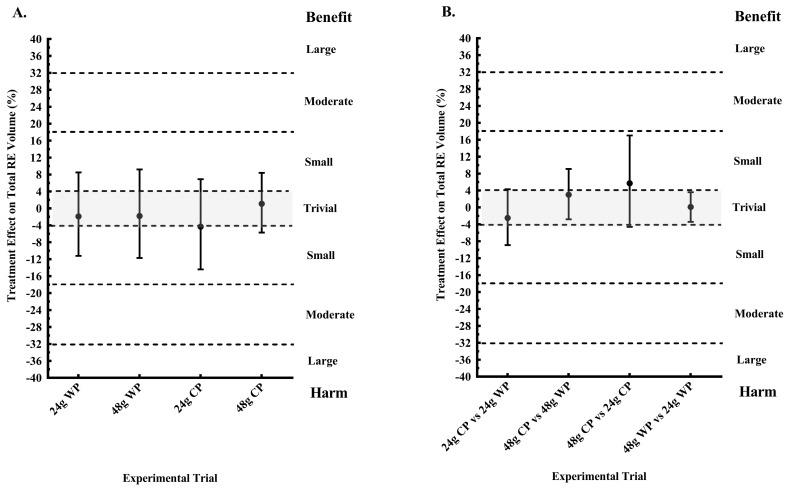
Treatment effects on total resistance exercise (RE) volume performed the morning after pre-sleep consumption of a single serving of 24 or 48 g whey protein (WP), 24 or 28 g casein protein (CP), compared (**A**) to a non-energetic placebo (PLA) and (**B**) other protein types and dose.

Total RE volume performed the morning after pre-sleep CP and WP, when compared to PLA are presented in Figure 2A. There were no clear effects of 24 g WP, 48 g WP, and 24 g CP. Only 48 g CP elicited a possibly trivial response in next morning RE training volume (+1.1 ± 7.0% mean effect, ES = 0.1; *p* = 0.779). Figure 2B displays the comparison between protein type and dose for total RE volume performed. When compared to 48 g WP, pre-sleep consumption of 48 g CP possibly (38% likelihood) increased RE volume (+3.0 ± 6.0% mean effect, ES = 0.13; *p* = 0.351), whilst the difference between 24 g CP and 24 g WP was unclear. Total RE volume performed was possibly (61% likelihood) greater after 48 g CP, compared to 24 g CP (+5.7 ± 10.8% mean effect, ES = 0.24; *p* = 0.440), but the comparison between 48 g WP and 24 g WP was likely trivial (+0.1 ± 3.5% mean effect, ES = 0.00; *p* = 0.899).

## 4. Discussion

The present study is the first to investigate the effect of pre-sleep consumption of a CP or WP supplement on next morning RE performance in physically active women. In addition, a primary aim of this study was to determine whether pre-sleep consumption of a low dose (24 g) and a high dose (48 g) of CP and WP increased next morning RMR, when compared to a PLA. The primary findings were that, when compared to not consuming any energy prior to sleep (PLA), only pre-sleep consumption of 48 g CP elicited a possibly trivial response in next morning RE training volume, a possibly and likely increase in energy expenditure as measured by VO_2_ and RMR, respectively; with an unclear effect on substrate utilization. However, 24 g CP and 48 g WP possibly and likely increased morning carbohydrate utilization, respectively, as determined by an increase in RER, but had unclear effects on RE performance. A secondary aim was to determine any differences between proteins (CP vs. WP) and dose (48 g vs. 24 g) of each protein on our outcome variables. Our findings suggested that 48 g of CP likely had a more favorable effect on morning RMR, and a possible benefit on RE performance, when compared to a similar dose of WP and 24 g of CP. 

Our observed findings of a likely acute increase in RMR in active individuals from pre-sleep nutrition, compared to not consuming energy, is consistent with previous studies that have used pre-sleep ingestion of single macronutrients [2] and chocolate milk [7]. However, a recently published study [14] did not find an increase in next morning RMR in mildly overweight men (BMI: 27.4 kg/m^2^) after consuming a pre-sleep skimmed milk snack, nor did they find a dose-response of low (10 g) vs. high (30 g) protein content on RMR and fat oxidation, compared to a non-energetic placebo. Similarly, in obese men, 30 g of CP did not lead to increased morning RMR, compared to a PLA [8]. One plausible reason for the difference between the findings by Lay et al. [14] and Kinsey et al. [8] with ours and those of others [2,7] is the type of participants involved in each study. It appears that acute, observable changes in next morning RMR are present in lean, physically active men [2] and women [7]. However, it is possible that these acute increases in RMR could extend to overweight and obese individuals through chronic pre-sleep feeding. Moreover, when combined with a regular exercise regimen, pre-sleep nutrition may confer favorable outcomes in overweight individuals, such as the increase in morning satiety. This was observed by Ormsbee et al. [32] in previously, sedentary obese women undergoing a 4-week pre-sleep nutrition combined with resistance exercise and high intensity interval training. In addition, the aforementioned studies have typically used a protein dose of 30 g and have not accounted for the body mass of participants when prescribing pre-sleep protein intake. Therefore, it is plausible that the commonly used 30 g dose may not be sufficient for heavier individuals and prescribing the amount of pre-sleep protein relative to body mass may be necessary. Thus, more research is needed to determine the optimal dose of pre-sleep protein intake to confer benefits to populations other than active individuals.

Nevertheless, a novel finding of the present study includes the likely increase in morning RMR after pre-sleep consumption of 48 g CP, compared to isoenergetic and isonitrogenous WP. This finding suggests that CP may be an ideal pre-sleep protein when consumed at doses greater than the previously studied 30 g. This may be true as comparisons in morning VO_2_, RMR, and RE performance, between 24 g of CP and WP, were unclear. However, considering our findings only revealed a possibly and likely benefit of 48 g CP on VO_2_ and RMR, respectively, compared to PLA, more research comparing the effects of the two protein types during the overnight period are needed. Interestingly, fat oxidation was likely greater after 48 g CP compared to 48 g WP, but fat oxidation was lower after consumption of 24 g CP when compared to 24 g WP. Previously, we reported that 30 g of pre-sleep CP did not inhibit next morning fat oxidation in physically active men, as 30 g of WP and carbohydrate did, when compared to a PLA [2]. Thus, it appears that at doses of 30 g or greater, CP may increase fat oxidation more than WP. This may also be due to the slow digesting nature of CP, resulting in a reported blunted insulin response when compared to WP [11]. Fat oxidation can be diminished by a rise in insulin secretion, and therefore may explain the reasons for the greater fat oxidation after 48 g CP, compared to 48 g WP. Therefore, in addition to the increases in RMR in the present study and those of our previous work [2], CP at doses greater than 30 g may be an ideal pre-sleep nutrient for utilizing fat as a fuel, during the overnight postprandial period. 

To date, only one other study [7] has investigated the effect of pre-sleep nutrition on performance during a next morning exercise bout, and our study is the first to examine the impact of pre-sleep nutrient intake on next morning RE performance. Active individuals seeking to exercise in the morning may forgo a pre-exercise morning meal or breakfast due to time constraints, fear of gastrointestinal distress, or aiming to increase fat utilization. Thus, our study sought to determine if pre-sleep protein consumption may be an adequate fueling strategy in lieu of an early morning pre-exercise meal or snack. We speculated that pre-sleep protein may improve overnight recovery from activities of daily living, and improve performance when exercise was performed the next morning. Previous research has shown that pre-sleep consumption of 40 g CP alone increases MPS, and increases MPS to a greater extent when a prior evening bout of RE was performed [15]. Although, our participants did not perform RE in the evening prior to pre-sleep consumption of 48 g CP, it is plausible that this dose was adequately digested and absorbed overnight, thus potentially having an impact on overnight recovery leading to the possibly trivial response in RE performance in the present study. Our findings suggest that among the four pre-sleep proteins consumed, only pre-sleep consumption of 48 g of CP in the absence of an evening exercise bout had a possibly trivial acute effect on next morning RE performance. Moreover, it should be noted that although our findings for 24 g CP, 24 WP, and 48 g WP were unclear, there were no likely harmful effects on RE performance from consuming these proteins prior to sleep. However, it is still unclear what the effect of chronic pre-sleep protein consumption will have on next morning exercise performance. 

The present study did have a few limitations which need to be addressed. First, the non-energy containing placebo we chose to use differed in serving size, texture, and taste and may have confounded RE performance. However, it was necessary to include a PLA that did not contain any energy to identify differences in our outcome variables, between pre-sleep nutrition and not consuming energy. Although this limitation may have impacted RE performance, which would have required motivation from the participants to perform, it likely had no effect on the objective metabolic measures. Secondly, we did not assess our female participants around the same phases of their menstrual cycle. Therefore, we were not able to control for any hormonal changes between trials, which may have affected our metabolic variables, but not likely the assessment of RE performance. Lastly, although there were no differences in self-reported dietary intake the 24 h period prior to each experimental trial, we did not provide a standardized diet during this period. Furthermore, it appears our participants may have underreported their energy intake, as the self-reported mean intake was less than the mean RMR measured in our study. Future studies should aim to control dietary intake 24–48 h prior to the experimental trials by providing a standardized meal, which meets each participant’s daily energy requirements to further determine the effects of pre-sleep nutrition. Furthermore, future studies may need to assess participants in a metabolic chamber overnight to elucidate the effect of the fast digesting WP on overnight metabolism.

## 5. Conclusions

Our study is the first to investigate the effect of pre-sleep consumption of two doses (24 and 48 g) of CP or WP, on next morning RE performance in physically active women. Our findings suggest that pre-sleep CP at a dose greater than the previously investigated 30 g, may be an effective strategy to maximize the overnight window of opportunity for the provision of nutrients for improving RMR, and possibly morning RE performance. Future, studies should investigate pre-sleep protein at a dose relative to body-weight, as well as the effect of long-term pre-sleep feeding.

## Figures and Tables

**Table 1:** Comparison of Proteins versus Non-Energetic Placebo on Metabolic Variables.

		Mean ± SD and Mean Effect Difference (%) ± 90% Confidence Interval		
Treatment		VO2 (mL/kg per min)	RMR kJ/day (kcal/day)	RER
24 g CP	Mean	3.62 ± 0.57	6268 ± 753 (1498 ± 180)	0.73 ± 0.04
	Mean effect	−3.0; ±7.6	−1.9; ±8.0	2.8; ±5.8
	Interpretation	Unclear	Unclear	Possibly increase
48 g CP	Mean	3.84 ± 0.42	6653 ± 628 (1590 ± 150)	0.70 ± 0.03
	Mean effect	2.7; ±5.8	4.0; ±4.8	−0.7; ±2.7
	Interpretation	Possibly increase	Likely increase	Unclear
24 g WP	Mean	3.59 ± 0.39	6180 ± 678 (1477 ± 162)	0.70 ± 0.02
	Mean effect	−3.8; ±3.2	−3.3; ±3.3	−1.3; ±3.6
	Interpretation	Unclear	Unclear	Unclear
	Mean	3.61 ± 0.32	6292 ± 444 (1504 ± 106)	0.73 ± 0.05
48 g WP	Mean effect	−3.4; ±3.2	−1.6; ±3.5	3.4; ±3.2
	Inference	Unclear	Unclear	Likely increase

Mean ± SD, mean effect difference, and inferences on variables the morning after pre-sleep consumption of a single serving of 24 or 48 g whey protein (WP), 24 or 28 g casein protein (CP), compared to a non-energetic placebo (PLA).

**Table 2:** Comparison of Metabolic Variables between Protein Type and Dose.

		Mean Difference (%); ± 90% Confidence Interval		
Comparison		VO_2_	RMR	RER
24 g CP vs. 24 g WP	Mean effect	0.9; ±8.6	1.4; ±8.7	4.2; ±3.2
	Interpretation	Unclear	Unclear	Likely increase
48 g CP vs. 48 g WP	Mean effect	6.3; ±7.0	5.7; ±5.2	−4.2; ±6.3
	Interpretation	Likely increase	Likely increase	Likely decrease
48 g CP vs. 24 g CP	Mean effect	5.9; ±8.4	6.1; ±7.8	−3.5; ±5.5
	Interpretation	Likely increase	Likely increase	Unclear
48 g WP vs. 24 g WP	Mean effect	0.5; ±5.8	1.8; ±5.9	4.8; ±3.4
	Interpretation	Unclear	Possibly increase	Very likely increase

Mean effect comparisons and inferences on variables the morning after pre-sleep consumption of a single serving of 24 or 48 g whey protein (WP), 24 or 28 g casein protein (CP).
